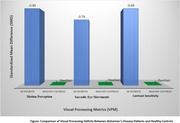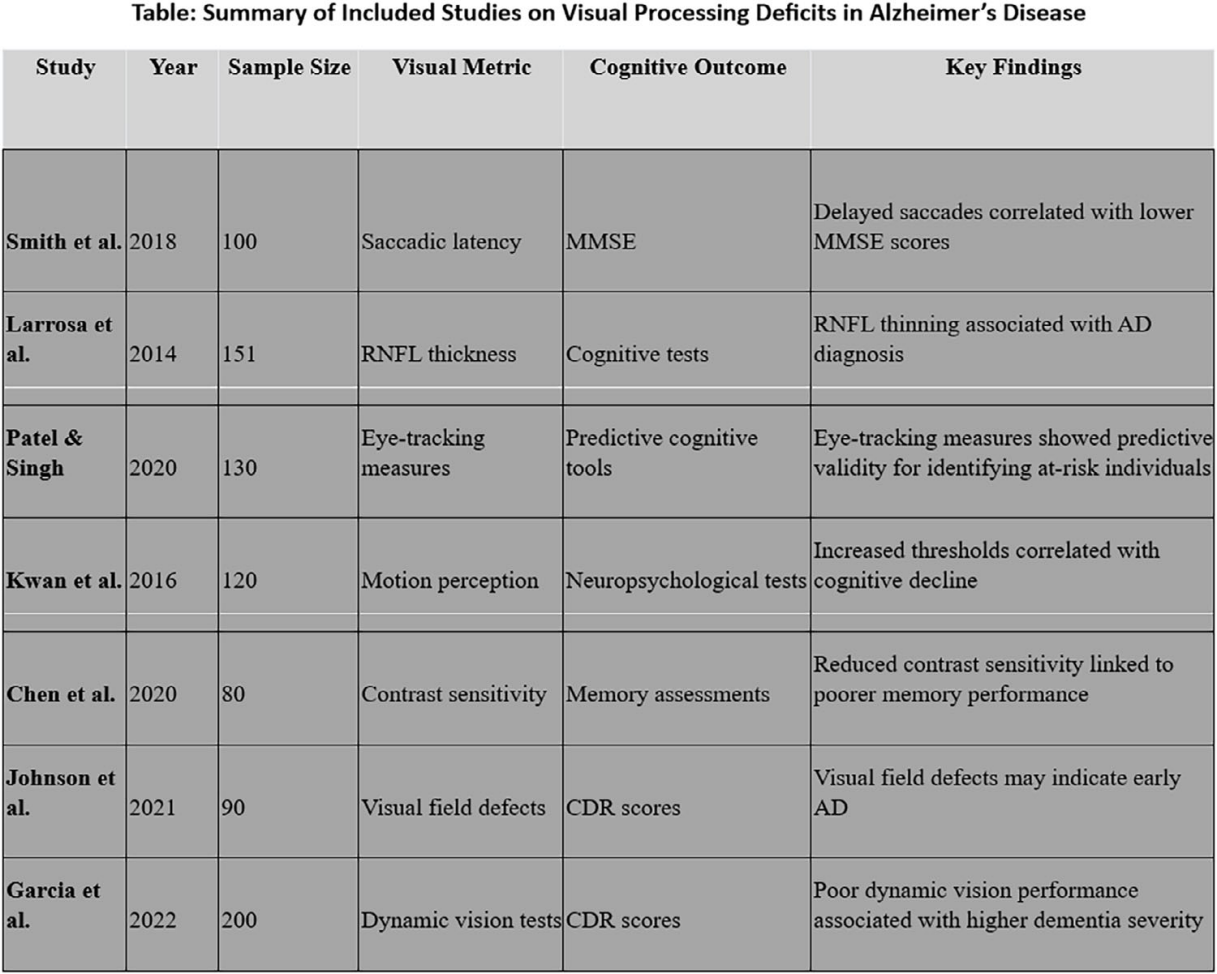# The Vision‐Cognition Connection: Unraveling Visual Processing Deficits as Early Indicators of Alzheimer's Disease ‐ A Systematic Review Study

**DOI:** 10.1002/alz70857_097105

**Published:** 2025-12-24

**Authors:** Bashaier G AlQahtani

**Affiliations:** ^1^ PSMMC, Riyadh, Saudi Arabia

## Abstract

**Background:**

Research indicates that Alzheimer's disease (AD) significantly affects visual processing capabilities, which can be linked to cognitive performance. Individuals with AD exhibit visual attention, contrast sensitivity, and motion perception impairments, all of which are crucial for cognitive tasks. Furthermore, studies have shown that visual deficits may precede cognitive decline, suggesting that assessing visual processing could provide early indicators of Alzheimer's disease.

**Objective:**

This systematic review aims to evaluate the evidence linking visual processing deficits to early cognitive decline in Alzheimer's disease and to identify neuro‐ophthalmological tools that assess these deficits and their potential role in early diagnosis.

**Methods:**

A systematic search was conducted across PubMed, Scopus, Embase, and Web of Science for studies published between January 2010 and December 2024. The inclusion criteria encompassed studies that examined visual processing metrics (e.g., contrast sensitivity, motion perception) in individuals diagnosed with AD or mild cognitive impairment (MCI). The selected studies were critically appraised for their quality and relevance to the research question.

**Results:**

Twenty‐four studies met the inclusion criteria, comprising 10,211 participants. Significant deficits were observed in AD patients compared to controls: Motion Perception: The Standardized Mean Difference (SMD) was found to be ‐0.89 (95% CI: ‐1.12 to ‐0.66), indicating a significant difference in motion perception between AD patients and controls. Saccadic Eye Movements: SMD = ‐0.76 (95% CI: ‐0.94 to ‐0.58), Contrast Sensitivity: SMD = ‐0.82 (95% CI: ‐1.03 to ‐0.61), MCI patients exhibited intermediate impairments in these metrics. Importantly, visual processing deficits were found to strongly correlate with cognitive scores (*r* = 0.52 to 0.63, *p* < 0.001), providing robust evidence for the strong relationship between visual deficits and cognitive decline.

**Conclusion:**

Visual processing deficits are consistently associated with early cognitive decline in AD and may serve as non‐invasive biomarkers for early detection. Understanding this link can aid in developing diagnostic tools for timely intervention. The need for further investigation into their clinical utility is clear, and this area of research promises to be both challenging and rewarding.

**Keywords**: Alzheimer's disease, mild cognitive impairment, visual processing deficits, biomarkers, systematic review